# Coalescence computations for large samples drawn from populations of time-varying sizes

**DOI:** 10.1371/journal.pone.0170701

**Published:** 2017-02-07

**Authors:** Andrzej Polanski, Agnieszka Szczesna, Mateusz Garbulowski, Marek Kimmel

**Affiliations:** 1 Institute of Informatics, Silesian University of Technology, ul. Akademicka 16, 44-100 Gliwice, Poland; 2 The Linnaeus Centre for Bioinformatics, Uppsala University, BMC, Uppsala, Sweden; 3 Systems Engineering Group, Silesian University of Technology, ul. Akademicka 16, 44-100 Gliwice, Poland; 4 Department of Statistics, Rice University, M.S. 138, 6100 Main Street, Houston, TX 77005, United States of America; Colorado State University, UNITED STATES

## Abstract

We present new results concerning probability distributions of times in the coalescence tree and expected allele frequencies for coalescent with large sample size. The obtained results are based on computational methodologies, which involve combining coalescence time scale changes with techniques of integral transformations and using analytical formulae for infinite products. We show applications of the proposed methodologies for computing probability distributions of times in the coalescence tree and their limits, for evaluation of accuracy of approximate expressions for times in the coalescence tree and expected allele frequencies, and for analysis of large human mitochondrial DNA dataset.

## Introduction

Coalescent theory [1, 2], widely used for statistical inference on genetic parameters and structures of evolving populations is a thoroughly studied area with many results published over decades. The classical coalescent model concerns a sample drawn from a population which has evolved with constant size over many generations in the past. For such a model many results concerning e.g., probability distributions of times in the coalescence tree [3, 4], expected ages [5, 6] and frequencies of mutations and recombinations [3, 4] were developed. Since majority of populations undergo changes in their size in the course of their evolution several authors developed coalescence computations for the case of time dependent population sizes, either by deriving analytical approaches [5, 7–9] or by using stochastic coalescence simulations [5, 10]. Other directions of developing coalescent modeling involve different scenarios of populations evolution, constant or undergoing expansions or bottlenecks, combined with possible inhomogeneity of their structures [11, 12], as well as different models of mutation, infinite size, infinite alleles, recurrent, stepwise. There are also several studies concerning coalescence modeling for populations under selection [13–15].

Emergence of large datasets resulting from contemporary sequencing technologies has drawn attention of researchers to problems in the coalescent theory arising in the situation where the coalescent sample size becomes large. There are several areas of analysis of such problems. Below we describe those of them, which are in the scope of our interest in the present paper. The first area is pursuing computations for existing algorithms for the case of large sizes of the coalescence tree. Many authors have pointed out (e.g., [7, 9, 16, 17] that some of the computational algorithms for coalescence modeling, published in the literature, are applicable only for relatively small sizes of samples, below 50. In view of availability of data of much larger sizes it is interesting and important to study and develop analogous or corresponding methodologies suitlable for larger sample sizes. The second area includes computing limits and/or growth/shrinkage patterns of distributions (expectations) of coalescence times and allele frequencies in the coalescence process. When the sample size tends to large values it becomes a natural and important question whether limits of distributions/values exist, how they can be computed and used in population and statistical genetics research. The third area includes developing large sample approximations. Large sample approximations usually have the form of simple analytical expressions and therefore may be very useful for (approximate) statistical inference. Large sample approximations also give a valuable support for intuitive understanding of mechanisms of evolution of large samples. Two types of approximations have been applied for large samples, deterministic and normal. Deterministic approximation is based on the fact that the coalescence process, which in principle has a stochastic nature, converges partly to a deterministic scenario when sample size goes to infinity [15, 18]. In the deterministic approach times in the coalescence process are represented by deterministic values. In the normal approximation approach times in the coalescence process are modeled (approximated) by normal distributions [16] on the basis of the fact that, under the constant population scenario, they are sums of many independent components [19].

The aim of the present paper is to show new results and conclusions in the three areas listed above. We derive our results by using new methods for computing exact probability distributions and expectations of times to coalescences for trees of arbitrary large sizes and for arbitrary scenarios of population time change. In previous publications [16, 20] such distributions and values were computed by using approximations or estimated by stochastic coalescence simulations. The proposed approach is based on deriving the inverse of the integral transform introduced in [7]. Further we derive the limit distribution of the time to most recent common ancestor, under different scenarios of population size change, which uses the gamma quotient formula for infinite products [21]. We show the following applications of the proposed approaches:

- Computing probability distributions and expectations of coalescence times for genealogies of large samples of DNA sequences, with high accuracy. In previous articles such distributions and expectations were estimated on the basis of coalescence simulations or approximate methods.
- Computing limit distributions of times to most recent common ancestor in the coalescene tree under different rates of population growth.
- Evaluation of accuracy of published large sample approximations [16] for times in the coalescence tree and expected allele frequencies.
- Estimation of rates of convergence of distributions of times in the coalescence tree to their limits.
- Fitting the exponential growth model to DNA polymorphisms data from the whole database of mitochondrial DNA for over 2000 individuals [22].

In the “Discussion” section we show some other possible applications of the derived results and some possible further directions of the research.

## Results

**Fig 1:**
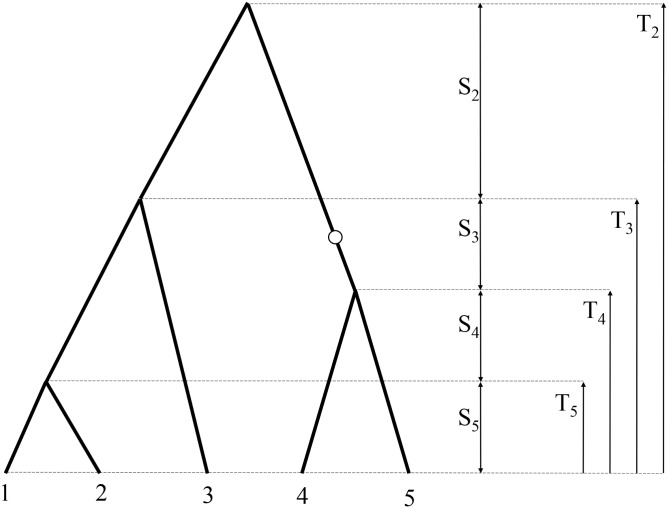
Coalescence tree and notation for ancestral history of a sample of *n* = 5 DNA sequences. Times to coalescence events are denoted by capital letters *T*, times between coalescences are denoted by capital letters *S*. A mutation event is marked by an open circle.

Results, which we show in this paper concern the past history of an *n*-sample (of DNA sequences) taken at present, as illustrated in Fig 1 where samples are numbered from 1 to *n* = 5. Time *t* is measured from the present to the past with the units defined by number of generations. We assume validity of the diffusion approximation [23], so *t* is a continuous variable. Coalescences are events of merging (joining) branches of the phylogenic tree of samples. Random coalescence times from sample of size *n* to sample of size *k* − 1 are denoted by *T*_*k*_, *k* = 2, 3…*n*, and their realizations by corresponding lower case letters *t*_*n*_, *t*_*n*−1_, …, *t*_2_, 0 < *t*_*n*_ < *t*_*n*−1_… < *t*_2_. Times between coalescence events are denoted by the capital and lower case letters *S*, *s*; in Fig 1 these times are denoted by *S*_5_, *S*_4_, …, *S*_2_. Apart from coalescnce times *T*_2_, …, *T*_*n*−1_, *T*_*n*_ and times between coalescence events *S*_2_, …, *S*_*n*−1_, *S*_*n*_ of special interest (e.g., [5, 7, 8, 16]) are also the time to the most recent common ancestor (*TMRCA*) and total length of branches in the coalescence tree (*TLBT*), defined as follows

$$\begin{array}{c} TMRCA=T_{2}, \end{array}$$ (1)

and

$$\begin{array}{c} TLBT=\sum_{k=2}^{n}kS_{k}=T_{2}+\sum_{k=2}^{n}T_{k}. \end{array}$$ (2)

During the DNA replication process mutation events occur along branches of the coalescence tree. In Fig 1 an exemplary mutation event is marked by an open circle. Consequently, sequences 4 and 5 have mutant alleles (bases), while sequences 1, 2 and 3—ancestral ones. We assume that the mutation process is described by a Poisson point process and that assumptions of the infinite sites model are satisfied (e.g., [5, 16, 24]). Allele frequencies corresponding to mutations depend on times in the coalescence tree *S*_*k*_ and on mutation intensity *μ*. Expected allele frequency, *f*_*nb*_, of mutation of type *b*, i.e., having *b* mutant bases versus *n* − *b* ancestral bases in the leaves of the coalescence tree (in Fig 1 *b* = 2, *n* − *b* = 3), is given by the following expression (e.g., [5, 16, 24])

fnb=μ(n-b-1)!(b-1)!(n-1)!∑k=2n(n-kb-1)k(k-1)E(Sk),1≤b≤n-1. (3)

Under the additional hypothesis that *μ* is close to zero, probability, *p*_*nb*_, that a randomly chosen mutation is of type *b* is given by the following expression [5]

$$\begin{array}{c} p_{nb}=\frac{f_{nb}}{\sum_{b}\;f_{nb}}=\frac{f_{nb}}{\mu E(TLBT)}. \end{array}$$ (4)

The effective size of the underlying population is assumed to be given by a deterministic function *N*(*t*), *t* ∈ [0, ∞). Two special cases of population size change (growth) scenarios are often researched, constant and exponential. The constant population size scenario is denoted as

$$\begin{array}{c} N\left(t\right)=N^{C}\left(t\right)=N_{0}. \end{array}$$ (5)

The exponential growth scenario is given by

$$\begin{array}{c} N\left(t\right)=N^{E}\left(t\right)=N_{0}\exp\left(-rt\right) \end{array}$$ (6)

where *r* is the exponent parameter. For exponential growth we also denote

$$\begin{array}{c} \rho =rN_{0}, \end{array}$$ (7)

and we call *ρ* the product parameter of the population exponential growth. With *r* = 0 the exponential scenario Eq (6) becomes the constant scenario Eq (5).

### Probability distributions of coalescence times

**Fig 2:**
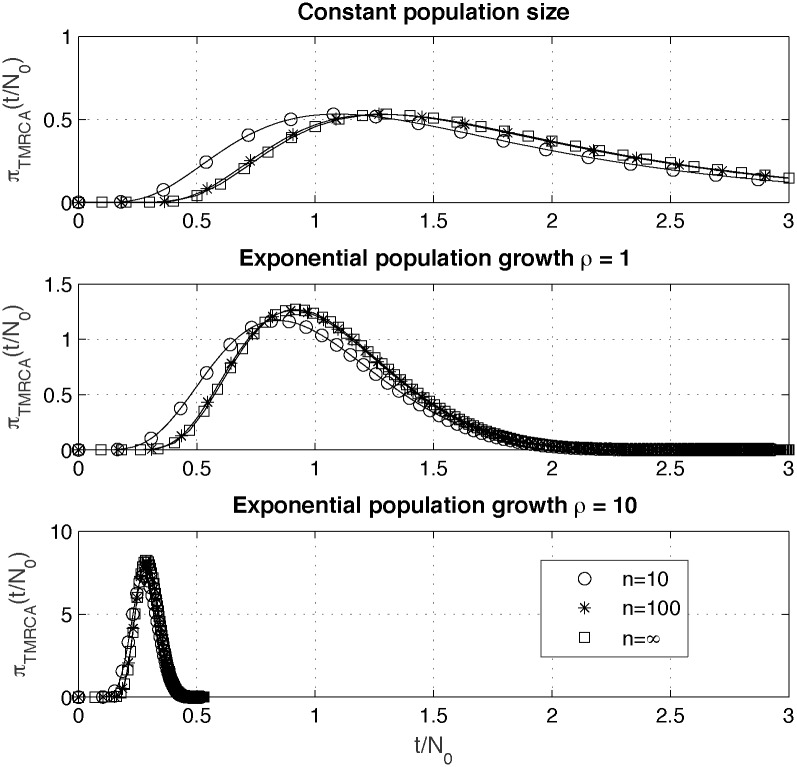
Probability density functions, $\pi_{TMRCA}\left(\frac{t}{N_{0}}\right)$, for different scenarios of populations size change, constant (upper plot) and exponentially growing with *ρ* = 1 (middle plot) and *ρ* = 10 (lower plot). Probability distributions are shown for different genealogy sizes *n* = 10, *n* = 100 and *n* = ∞ (limit distribution).

In this subsection we present results concerning probability distributions of times in the coalescence tree, which according to our best knowledge were not published before. We obtain them by using the methods described in subsections “Inversion of the integral transform” and “Limit distributions” of the “Methods” section. In Fig 2 we show probability distributions of *TMRCA*, $\pi_{TMRCA}\left(\frac{t}{N_{0}}\right)$, for genealogy sizes *n* = 10, *n* = 100 and *n* = ∞, for different scenarios of populations size change, constant (upper plot) and exponentially growing with *ρ* = 1 (middle plot) and *ρ* = 10 (lower plot). Convergence of probability density functions of *TMRCA* to the limit distribution, derived in subsection “Limit distributions”, is rather fast. One can observe that time scale change related to exponential scenario of population growth with increasing *ρ* results in probability distribution of *TMRCA* with increasing similarity to normal distribution.

The result published by [19] states that probability distributions of times *T*_*k*_ in the middle of the coalescence tree converge to normal distribution when *n* → ∞. Using our expressions from subsection “Inversion of the integral transform” of the “Methods” section, we have numerically studied the rate of this convergence by computing skewness coefficients *γ*(*T*_*k*_),

$$\begin{array}{c} \gamma \left(T_{k}\right)=E\left[{\left(\frac{T_{k}-E\left(T_{k}\right)}{Std(T_{k})}\right)}^{3}\right] \end{array}$$ (8)

for distributions of times *T*_*k*_ when the index *k* changed from top to the bottom of the coalescence tree. Values of skewness coefficient allow for estimating departure of the distribution of interest from normality.

**Fig 3:**
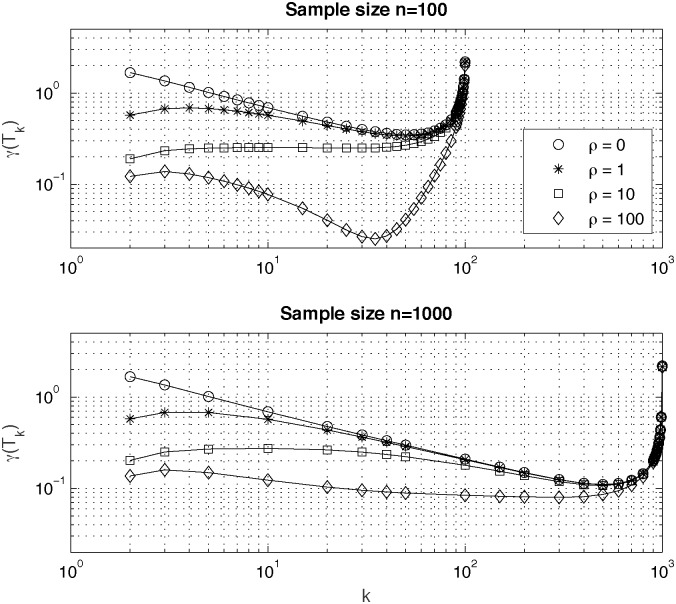
Values of skewness coefficient *γ*(*T*_*k*_) of probability distributions of times in the coalescence tree computed for different genealogy sizes, *n* = 100 (upper plot) and *n* = 1000 (lower plot) and for different scenarios of population size change constant (*ρ* = 0) and exponentially growing with *ρ* = 1, *ρ* = 10 and *ρ* = 100.

Plots of skewness coefficient *γ*(*T*_*k*_) for different genealogy sizes, *n* = 100 (upper plot) and *n* = 1000 (lower plot) and for different scenarios of population size change constant (*ρ* = 0) and exponentially growing with *ρ* = 1, *ρ* = 10 and *ρ* = 100 are shown in Fig 3.

From the plots in Fig 3 one can see that skewness of probability distributions of times *T*_*k*_ decrease for increasing values of the product parameter *ρ*. For all scenarios, constant and exponential with different *ρ*, one observes a sharp increase of values of skewness coefficient *γ*(*T*_*k*_) in the fourth quartile of the range of values of the coalescence tree index *k*.

### Accuracy of approximate formulae for expectations of coalescence times

Large sample approximations for probability distributions and expectations of coalescence times are very useful due to both their simple forms, and applicability to samples of arbitrary large size. Chen and Chen, (2013) [16], derived large sample approximations for expected coalescence times, $E(T_{k}^{E})$, *ETMRCA*, *ETBLT* for the case of exponential growth of population underlying the coalescence process. An important issue for applications of Chen and Chen’s approximate formulae (“Methods” section, Eqs (26)–(28), is how accurately they approximate exact values. Chen and Chen (2013) [16] in their Figure 5 show plots, which demonstrate accuracy of their approximations of *ETMRCA* Eq (27) and *ETBLT* Eq (28). However, estimation of accuracy of approximation of *ETMRCA* (upper plot “A” in Figure 5 in Chen and Chen, (2013) [16] is based only on simulations, which reduces precision of estimation. The lower plot “B” in Figure 5 in Chen and Chen, (2013) [16] shows good accuracy of approximation Eq (28). However, one can examine accuracy only in qualitative terms.

Here we precisely evaluate accuarcy of Chen and Chen’s approximations by using the approach presented in the “Methods” section. This approach is justified by results of the numerical study which proves that for sample sizes of orders of hundreds of thousands relative accuracy is better than 10^−6^ (see the Methods section).

**Fig 4:**
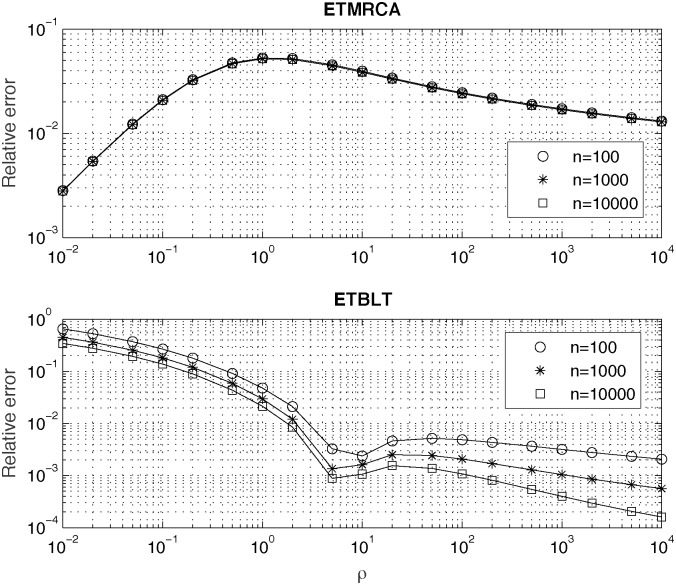
Relative errors of approximations for *ETMRCA* (upper plot) and *ETBLT* (lower plot) proposed by Chen and Chen (2013) [16].

In Fig 4 we show plots of relative errors of Chen and Chen’s, (2013) [16] approximations (“Methods” section, Eqs (27) and (28) for *ETMRCA* and *ETBLT* computed by using our expressions given in “Methods” section, Eqs (22)–(25). It is easily seen, that relative error for both *ETMRCA* and *ETBLT*, for a given sample size *n* depends only on the value of the product parameter of the population growth *ρ* Eq (7). When computing approximate *ETBLT* one needs to replace the value on the right hand side of Eq (28) by its limit, 2*N*_0_, in the case when *n* = 2*rN*_0_ = 2*ρ*. As seen from upper and lower plots in Fig 4 relative approximation errors of *ETMRCA* and *ETBLT* show quite complicated, nonlinear dependence on *ρ*. Relative error committed when using Chen and Chen’s approximation approximation for *ETMRCA* (“Methods” section, Eq (27)) is of order of percents. For *n* > 100 this error practically does not depend on *n*, which is consistent with fast convergence of distribution of *TMRCA* (shown in Fig 2). Relative error committed when using Chen and Chen’s approximation for *ETBLT* (“Methods” section, Eq (28)) increases for small values of *ρ* and decreases for large *ρ* and for large sample sizes *n*. For values of *ρ* > 10 and for sample sizes *n* > 100 accuracy of approximation Eq (28) is very good, of the order of 10^−3^ or better, consistently to results already shown in [16]).

In their Table 1 Chen and Chen (2013) [16] show values of asymptotic approximations of expectations and standard deviations of coalescence times. In order to evaluate accuracy of these approximations they compare them to averages computed over stochastic simulations, which results in committing error resulting from random variation in simulations. Below in our Table 1 we have reproduced one part of Chen and Chen’s (2013) [16] Table 1, corresponding to sample size *n* = 800. In our Table 1 we have replaced estimates of expectations and standard deviations of coalescence times obtained by simulations by their exact values computed with the use of our algorithm described in subsection “Inversion of the integral transform” of the “Methods” section. Chen and Chen (2013) [16] use index named *m* to number coalescence times. Our index used for numbering coalescence times is *k*. Due to different notation conventions these indexes must be shifted by 1 in order to obtain corresponding results.

**Table 1:** Comparison of exact expectations and standard deviations of times to coalescence *T*_*k*_ to their asymptotic approximations proposed by Chen and Chen (2013) [16], for *n* = 800.

*r*	*k*	Mean *T*_*k*_			Standard deviation of *T*_*k*_		
		Exact	Asymptotic	Bias%	Exact	Asymptotic	Bias%
0.001	6	6647.928	6679.599	0.476	250.612	258.485	3.141
0.001	11	5964.931	5981.414	0.276	181.193	184.235	1.678
0.001	51	4327.067	4330.733	0.085	85.654	85.933	0.326
0.001	201	2771.310	2772.589	0.046	50.608	50.631	0.045
0.001	401	1790.748	1791.759	0.057	45.001	45.005	0.009
0.001	796	30.870	30.962	0.299	13.556	13.635	0.581
0.005	6	1651.262	1657.606	0.385	50.175	51.749	3.136
0.005	11	1514.458	1517.766	0.218	36.314	36.922	1.674
0.005	51	1185.169	1185.918	0.063	17.314	17.369	0.317
0.005	201	865.864	866.147	0.033	10.656	10.659	0.030
0.005	401	651.350	651.619	0.041	10.389	10.386	0.030
0.005	796	28.849	29.206	1.243	11.891	12.153	2.204
0.01	6	894.933	898.105	0.354	25.091	25.878	3.137
0.01	11	826.518	828.172	0.200	18.167	18.465	1.670
0.01	51	661.765	662.141	0.057	8.669	8.696	0.315
0.01	201	501.585	501.728	0.029	5.363	5.365	0.032
0.01	401	393.043	393.183	0.036	5.297	5.295	0.034
0.01	796	26.796	27.343	2.043	10.361	10.699	3.262

Comparing values of biases in our Table 1 to their counterparts in Chen and Chen’s (2013) [16] Table 1 one can see that Chen and Chen’s (2013) [16] were able to estimate magnitudes of biases, however it was not possible for them to compute exact values.

### Accuracy of approximate formulae for expected allele frequencies

We evaluate accuracy of Chen and Chen’s (2013) [16] method for approximate computation of expected allele frequencies based on the idea of replacing expected times in coalescence process with underlying exponentially growing population, by their approximations (“Methods” section, Eq (26)). In Chen and Chen’s (2013) [16] Figure 6 one can observe that approximate method proposed by Chen and Chen (2013) [16] leads to estimates, which properly reflect patterns of change of expected allele frequencies. However, this obsevation can be done only qualitatively.

**Fig 5:**
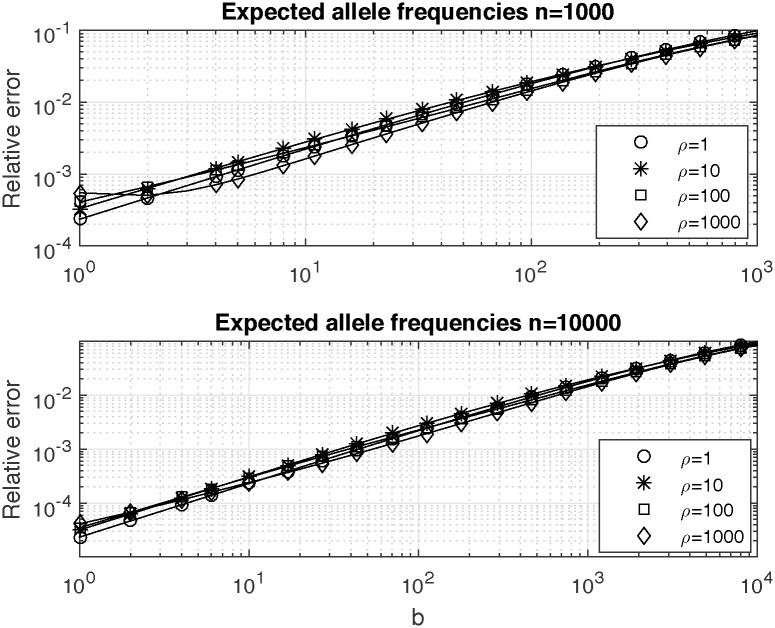
Relative errors of expected allele frequencies *q*_*nb*_ versus allele type *b* for two values of genealogy size *n* = 1000 (upper plot) and *n* = 10000 (lower plot) for different values of the product parameter of the population growth *ρ* = 1, *ρ* = 10, *ρ* = 100 and *ρ* = 1000.

In Fig 5 we show values of relative errors of expected allele frequencies *q*_*nb*_ versus allele type *b* for two values of genealogy size *n* = 1000 (upper plot) and *n* = 10000 (lower plot) for different values of the product parameter of the population growth *ρ* = 1, *ρ* = 10, *ρ* = 100 and *ρ* = 1000. Relative error shows nonlinear behavior with respect to changes in *ρ*. For the range of values of *ρ* depicted in Fig 5 values of the relative error are small (of the order of 10^−4^—10^−3^) for low values of *b* and grow to about 10% for high values of *b*. Since accurate computation of expected frequencies for alleles corresponding to low values of *b* is more important than for those corresponding to high values of *b*, Chen and Chen’s (2013) [16] approximation seems useful for many applications.

### Analysis of mitochondrial DNA dataset

The last result, which we show is the analysis of human mitochondrial DNA polymorphisms from the Human Mitochondrial Genome mtDB database [25]. The mtDB database contains in total 3857 polymorphic sites quantified in 2704 individuals. For our analysis we have chosen only a subset of 3857 polymorphic sites in mtDB, namely those sites whose status was determined for all 2704 individuals and which were diallelic. There were 3213 such segregating sites. Table 2 shows their allelic frequencies.

**Table 2:** Statistics of segregating sites in mtDNA data from Human mtDNA database [22]. Elements in *b* are possible numbers of copies of the rare allele, and elements in *c*_*k*_ are numbers of segregating sites in the sample that have the number of copies of the rare allele equal *b*.

*b*	*c*_*k*_	*b*	*c*_*k*_	*b*	*c*_*k*_	*b*	*c*_*k*_	*b*	*c*_*k*_
1	1231	28	10	55	5	87	1	156	1
2	542	29	5	56	2	88	1	174	1
3	298	30	5	57	1	89	1	176	1
4	170	31	8	58	4	90	1	204	1
5	149	32	10	59	1	91	1	213	1
6	95	33	7	60	3	94	1	218	1
7	66	34	5	61	4	95	2	234	1
8	67	35	8	62	3	96	3	235	1
9	35	36	9	63	2	98	2	244	1
10	33	37	8	64	1	104	1	264	1
11	28	38	2	65	3	110	1	272	1
12	21	39	8	66	1	111	1	299	1
13	17	40	1	67	1	127	1	347	2
14	24	41	3	68	1	128	1	390	1
15	16	42	5	69	2	129	2	444	1
16	22	43	5	70	1	131	2	505	1
17	15	44	7	72	1	132	2	550	1
18	19	45	5	74	1	133	1	604	1
19	13	46	1	76	1	134	1	610	1
20	17	47	6	77	2	135	1	720	1
21	10	48	4	78	1	138	2	724	1
22	13	49	1	79	1	139	1	777	1
23	14	50	1	81	1	144	1	867	1
24	8	51	3	83	3	147	3	933	1
25	5	52	2	84	4	149	3	943	1
26	15	53	2	85	3	150	1	944	1
27	13	54	1	86	6	152	1		

**Fig 6:**
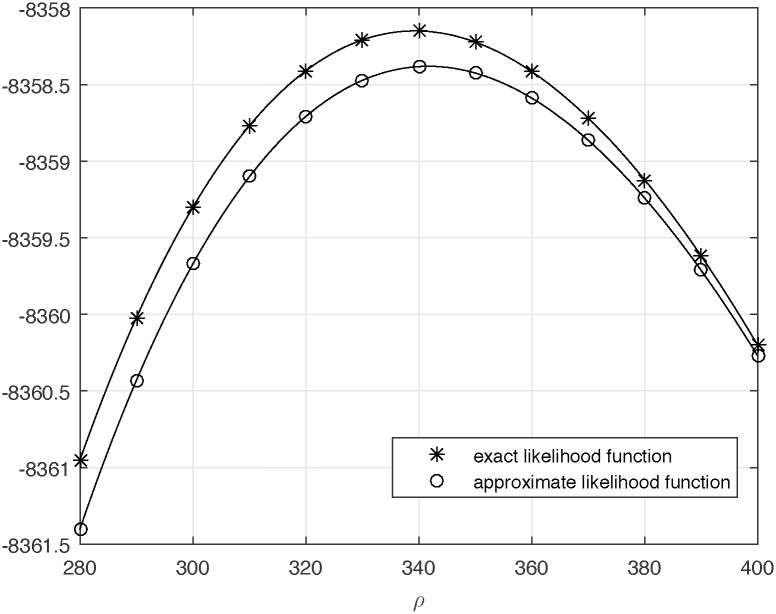
Log-likelihood curves for the exponential model of population growth for data on segregating sites from the mtDB database [25]. Each segregating site from Table 2 was treated as a separate SNP. The curve marked with asterisks shows the exact log likelihood function and the one marked with open circles is the approximate log likelihood function. The maximum of the exact log likelihood function is attained at ${\hat{\rho }}_{exact}=339.3$ and the maximum of the approximate log likelihood function is attained at ${\hat{\rho }}_{approx}=341.7$.

We have fitted the model of exponential growth for the data given in Table 2. Analogously to [8] we treated each segregating site as a separate SNP. The model fit is based on maximizing the likelihood function defined in [8] (Eq (24)). In Fig 6 we present plots of log likelihood functions versus exponential growth product parameter *ρ*, obtained with the use of exact method (marked with asterisks) and with the use of approximate expectations of coalescence times proposed by Chen and Chen (2013) [16] (marked with open circles). One can see that plots are quite close one to another, consistently to results presented in subsection “Accuracy of approximate formulae for expected allele frequencies”. Maximum likelihood estimate obtained by using the exact method is ${\hat{\rho }}_{exact}=339.3$ and the estimate obtained by using the approximate method is ${\hat{\rho }}_{approx}=341.7$. Additionally, we used Hudson’s program “ms” [26] to perform 1000 coalescence simulations, with appropriate parameters, which allowed us to estimate 95% confidence interval as 285 < *ρ* < 403. Similar estimates can be obtained on the basis of the likelihood ratio statistics [8]. The obtained values and bounds of confidence intervals, fit into the range of values (50–500) of exponential growth product parameter of human population, which we estimated in [8] on the basis of different datasets.

## Discussion

In this paper we evaluate the accuracy of the approximations for times in the coalescence tree and expected allele frequencies as proposed by [16] and we compute the probability distributions of times in the coalescence tree and their limits. We also use Human Mitochondrial Genome mtDB database to present a comparison of exact versus approximate log likelihood function for solving the inverse problem of estimating population size history from observed allele frequencies [18, 20, 24].

The presented resuts are based on new approaches described in the “Methods” section. We propose new methods for coalescence computations for large sample sizes, based on inverting the integral transform defined in [7] (“Methods” section, Eq (12)) and on using analytical expressions for infinite products for computing limit distributions. Both the integral transform Eq (12) and its inverse Eq (29) use techniques well known in statistical genetics—the Laplace (Fourier) transformation and coalescence time scale change. However, combining them together allows for deriving new results, unavailable in the previous literature.

Methodologies for efficient computation of probability distributions and expectations of coalescence times for large sample sizes, presented in the “Methods” section of this paper, can lead to many further applications. The inverse transform Eq (29) can be generalized to higher dimensions. Two-dimensional generalization of Eq (29) can be used for computing second-order moments of coalescence times [27] for large sample sizes. Methodology for computing second-order moments of coalescence times can be useful e.g., for analyzing statistics of triallelic DNA loci [28].

In this paper, by large sample sizes *n* we understand numbers comparable to the present throughput capabilities of experimental techniques for DNA sequencing, i.e. thousands of people, with data publically available in databases in 1000 Genomes Project [29] or mtDB [25]. The sample size is going to increase to hundreds of thousands or even millions in short order, with ongoing projects including UK10K (about 10 thousand human genomes) and the Million Veteran Program (about 1 million human genomes). The computational methods proposed in this paper are likely to be relevant to many aspects of statistical analyses of these datasets.

Sequencing data for even larger number of cell samples are already available in the cancer genomics TCGA database [30]. Cancer tissue is an evolving population of cancer cells with diversity increasing as the tumour advances in development, and using coalescence modeling for the analysis of cancer genomics data [17, 31] is of great interest and possibly of significant practical importance. Cell count in cancer tissues exceeds bilions, and biopsies include upwards of millions of cells [32]. However, developing algorithms for coalescence analyses of cancer genomics sequencing data requires addressing not only the problem of large sample size but also numerous additional issues specific to that type of data. Typical sequencing cancer genomics data include reads obtained from a mixture rather than from separate cancer cells, which calls for the development of integrative approaches combining large sample coalescence modeling with ascertainment models (e.g., [33]). Another concern would be the presence of mutational events such as chromosomal duplications, the loss of heterozygosity and rearangements [31], which interfere with the point mutation processes. Additionally, point mutations seen in the cancer sequencing samples are classified as either driver or passenger, which is related with their roles in the selection mechanisms in the carcinogenesis process. Driver mutations are defining new clones creating cancer cell population subdivisions, leading to the need for further model refinement. Some of the problems listed above are possible topics of present studies on coalescence modeling methods for applications in cancer genomics.

## Methods

In the case of the evolutionary scenario with the constant population size times between coalescence events, $S_{n}^{C},\;S_{n-1}^{C},\ldots ,\;S_{2}^{C}$, are mutually independent random variables, each distributed exponentially, with expectations (e.g., [5])

E(SkC)=N0(k2),k=2,3,...,n. (9)

For the case of constant population size one can obtain analytical expressions for expected allele frequencies $f_{nb}^{C}$ and probabilities $p_{nb}^{C}$ [5, 34].

In the general case of the population size history given by a function *N*(*t*), times between coalescences, *S*_2_, …, *S*_*n*−1_, *S*_*n*_, are not independent. Joint probability density function of the distribution of times *T*_2_, …*T*_*n*−1_, *T*_*n*_ can be computed by using the following expression [35].

p(t2,...,tn-1,tn)=∏j=2n(j2)N(tj)exp(-∫tj+1tj(j2)dσN(σ)) (10)

where 0 = *t*_*n*+1_ < *t*_*n*_ < *t*_*n*−1_… < *t*_2_, and (j2) is the binomial symbol. Marginal distributions of times *T*_2_, …, *T*_*n*−1_, *T*_*n*_, denoted by *π*_*T*2_(*t*), …, *π*_*Tn*−1_(*t*), *π*_*Tn*_(*t*) follow from multiple integrations of the above formula Eq (10).

A method for computing marginal distributions, *π*_*T*2_(*t*), …, *π*_*Tn*−1_(*t*), *π*_*Tn*_(*t*), based on combining the time scale change

$$\begin{array}{c} \tau =g\left(t\right)=\int_{0}^{t}\frac{d\sigma }{N(\sigma )} \end{array}$$ (11)

with the technique of integral transformations was proposed in [7]. The proposed integral transform ϒ{.} with the underlying function *N*(*t*) was defined as follows

$$\begin{array}{c} P\left(s\right)=ϒ\left\{\pi \left(t\right)\right\}=E\left[\exp\left(-s\int_{0}^{t}\frac{d\sigma }{N(\sigma )}\right)\right]=\int_{0}^{\infty }\pi \left(t\right)\exp\left(-s\int_{0}^{t}\frac{d\sigma }{N(\sigma )}\right)dt. \end{array}$$ (12)

Application of ϒ transformation led to analytical expressions for ϒ transforms of marginal distributions

ϒ{πTk(t)}=∏j=kn(j2)s+(j2), (13)

and to expressions for marginal distributions

$$\begin{array}{c} \pi_{Tk}\left(t\right)=\sum_{j=k}^{n}A_{jk}^{n}q_{j}\left(t\right)\;k=2,3,...,n, \end{array}$$ (14)

where coefficients $A_{jk}^{n}$ followed from partial fraction expansion of the product in Eq (13) (see Eq (7) in [7]), and

qj(t)=(j2)N(t)exp(-∫0t(j2)dσN(σ)). (15)

The probability distribution, *q*_*j*_(*t*), in the above formula Eq (15) is the distribution of the time to the first coalescence event in the sample of size *j*. The formula Eq (14) was also independently derived by Zivkovic and Wiehe (2008) [27] by repeated integration and using mathematical induction.

By using Eqs (14) and (15) one can compute expectations of times to coalescences *E*(*T*_*n*_), *E*(*T*_*n*−1_), …, *E*(*T*_2_)

$$\begin{array}{c} E\left(T_{k}\right)=\sum_{j=k}^{n}A_{jk}^{n}e_{j}\;k=2,3,...,n, \end{array}$$ (16)

where

$$\begin{array}{c} e_{j}=\int_{0}^{\infty }tq_{j}\left(t\right)dt, \end{array}$$ (17)

are expected times to the first coalescence event in a sample of size *j*. Expressions for expectations of times *T*_*n*_, *T*_*n*−1_, …, *T*_2_ Eq (16) can be used for computing expectations *E*(*S*_2_), …, *E*(*S*_*n*−1_), *E*(*S*_*n*_) and *ETLBT*. Consequently, formula (Eq (3)) can be applied for computing allele frequencies.

For the case where *N*(*t*) follows the exponential scenario, *N*(*t*) = *N*^*E*^(*t*) Eq (6) time scale change in Eq (11) becomes

$$\begin{array}{c} \tau =g_{E}\left(t\right)=\frac{1}{rN_{0}}\left(\exp\left(rt\right)-1\right) \end{array}$$ (18)

and *q*_*j*_(*t*) in Eq (15) becomes

qjE(t)=(j2)N0exp[rt+(j2)rN0(1-exp(rt))]. (19)

Analogously to Eqs (16) and (17), by using Eqs (14) and (19) one can compute expectations for the exponential scenario

$$\begin{array}{c} E\left(T_{k}^{E}\right)=\sum_{j=k}^{n}A_{jk}^{n}e_{j}^{E} \end{array}$$ (20)

where $e_{j}^{E}$ are expectations of times with probability distributions given in Eq (19), equal to [8, 10]

$$\begin{array}{c} e_{j}^{E}=e_{j}^{E}\left(N_{0},r\right)=-\frac{\exp[{(}_{2}^{j}){\left(rN_{0}\right)}^{-1}]}{r}Ei\left[-{(}_{2}^{j}){\left(rN_{0}\right)}^{-1}\right]. \end{array}$$ (21)

In the above *Ei* denotes the exponential integral, $\text{Ei}\left(-\mu \right)=-\int_{1}^{\infty }\left[\exp\left(-\mu x\right)/x\right]dx$, Re(*μ*) > 0, ([36], 3.351.5). Eq (20) can be used for computing $E\left(S_{2}^{E}\right),...,\;E\left(S_{n-1}^{E}\right),\;E(S_{n}^{E}$), *ETMRCA*^*E*^, *ETLBT*^*E*^ and, substituted in Eq (3), for computing allele frequencies.

As we have already mentioned in the introduction section, many authors [7–9, 16, 17] have reported that expressions for probability distributions and expectations of times, and for allele frequencies of mutations for the general case of population size history *N*(*t*) are applicable only for small sample sizes *n* < 50, due to the fact that coefficients $A_{jk}^{n}$ very quickly diverge to very large numbers with alternating signs when *n* increases.

The approach based on application of combinatorial identities and methods of summing hypergeometric series given in [7, 8], which allows for obtaining numerically stable expressions for *ETMRCA*, *ETLBT* and expected allele frequencies *f*_*nb*_, applicable for large values of *n*. These expressions have the following forms

$$\begin{array}{c} ETMRCA=\sum_{j=2}^{n}\left(2j-1\right)\frac{n!(n-1)!}{(n+j-1)!(n-j)!}{\left(-1\right)}^{j}e_{j}, \end{array}$$ (22)

$$\begin{array}{c} ETLBT=\sum_{j=2}^{n}\left(2j-1\right)\frac{n!(n-1)!}{(n+j-1)!(n-j)!}\left[1+{\left(-1\right)}^{j}\right]e_{j} \end{array}$$ (23)

and

$$\begin{array}{c} f_{nb}=\mu \sum_{j=2}^{n}W_{bj}^{n}e_{j},\;b=1,2,...,n-1. \end{array}$$ (24)

Coefficients $W_{bj}^{n}$ in Eq (24) are given by the recursion below

$$\begin{array}{c} W_{b2}^{n}=\frac{6}{\left(n+1\right)},\;W_{b3}^{n}=30\frac{\left(n-2b\right)}{\left(n+1)(n+2\right)}, \end{array}$$

$$\begin{array}{c} W_{b,j+2}^{n}=-\frac{\left(1+j)(3+2j)(n-j\right)}{j(2j-1)(n+j+1)}W_{bj}^{n}+\frac{\left(3+2j)(n-2b\right)}{j(n+j+1)}W_{b,j+1}^{n}, \end{array}$$ (25)

*j* = 2, 3, …, *n* − 2. In Eqs (22)–(24) *e*_*j*_ denote expected times to the first coalescence event in a sample of size *j* given in Eq (17). By using expressions Eqs (9), (15)–(17) and (21) one can compute expectations *e*_*j*_ and further *ETMRCA*, *ETLBT* and *f*_*nb*_ for any scenario of population size change, constant ($e_{j}^{C}$), given by generally defined function *N*(*t*) (*e*_*j*_) and exponential ($e_{j}^{E}$).

Expressions Eqs (22)–(25) were used by several authors for computing exact values of expected times *ETMRCA*, *ETLBT* and expected allele frequencies, e.g., or for studying properties of coalescence process [37], for studies on pupulation size histories [38] and for comparisons between exact and approximate methods [16].

By applying time scale change *g*^−1^(*τ*), given in Eq (36) Chen and Chen, (2013) [16] have obtained the following approximation of expected coalescence times for the exponential growth scenario

$$\begin{array}{c} E\left(T_{k}^{E}\right)≃\frac{1}{r}\ln\left[2rN_{0}\left(\frac{1}{k-1}-\frac{1}{n}\right)+1\right], \end{array}$$ (26)

and

$$\begin{array}{c} ETMRCA^{E}=E\left(T_{2}^{E}\right)≃\frac{1}{r}\ln\left[2rN_{0}\left(1-\frac{1}{n}\right)+1\right]. \end{array}$$ (27)

By integrating over time expectation of the pure death process describing merging of branches of the coalescence tree Chen and Chen, (2013) [16] have also derived the following approximation for expected total length of branches in the coalescence tree under exponential scenario *ETBLT*^*E*^

$$\begin{array}{c} ETBLT^{E}≃\frac{2nN_{0}\ln\frac{2rN_{0}}{n}}{2rN_{0}-n}. \end{array}$$ (28)

Finally Chen and Chen, (2013) [16] have proposed to substitute approximate expectations of coalescence times Eq (26) in expression Eq (3) to obtain approximate expected allele frequencies in the coalescence process with the underlying exponential population growth.

### Inversion of the integral transform

The limitation of applicability of computations based on combinatorial identities and summing hypergeometric series is that they can only be used for expectations *ETMRCA*, *ETLBT* and for expected allele frequencies *f*_*nb*_. Computing probability distributions of times *T*_*k*_ is not possible.

In this subsection we present a new approach, which allows for computing distributions and expectations of times to coalescence events, *T*_2_, …, *T*_*n*−1_, *T*_*n*_, with (theoretically) arbitrary accuracy, applicable for large genealogies. The approach is based on construction of the transformation inverse to Eq (12). The inverse of Eq (12), denoted by ϒ^−1^{.}, has the following form

$$\begin{array}{c} \pi \left(t\right)={ϒ}^{-1}\left\{P\left(s\right)\right\}=\frac{1}{N(t)}\frac{1}{2\pi i}\int_{c-i\infty }^{c+i\infty }P\left(s\right)\exp\left(s\int_{0}^{t}\frac{d\sigma }{N(\sigma )}\right)ds. \end{array}$$ (29)

In Eq (29) *c* is a suitably chosen constant and $i=\sqrt{-1}$. The above formula is constructed by analogy to the inverse of the Laplace transform, Mellin—Fourier integral [39]. Since *π*(*t*) is a density function of the probability distribution we can set *c* = 0, *s* = *iω* and replace Eq (29) by

$$\begin{array}{c} \pi \left(t\right)={ϒ}^{-1}\left\{P\left(i\omega \right)\right\}=\frac{1}{N(t)}\frac{1}{2\pi }\int_{-\infty }^{\infty }P\left(i\omega \right)\exp\left(i\omega \int_{0}^{t}\frac{d\sigma }{N(\sigma )}\right)d\omega . \end{array}$$ (30)

Verification that ϒ^−1^[ϒ(*π*(*t*))] = ϒ^−1^[*P*(*s*)] = *π*(*t*) is straightforward, since either Eq (29) or Eq (30) can be understood as a two-step procedure. The first step is the inverse Laplace

$$\begin{array}{c} \pi^{C_{0}}\left(\tau \right)=\frac{1}{2\pi i}\int_{c-i\infty }^{c+i\infty }P\left(s\right)\exp\left(s\tau \right)ds \end{array}$$ (31)

or inverse Fourier transform

$$\begin{array}{c} \pi^{C_{0}}\left(\tau \right)=\frac{1}{2\pi }\int_{-\infty }^{\infty }P\left(i\omega \right)\exp\left(i\omega \tau \right)d\omega \end{array}$$ (32)

of *P*(*s*) = ϒ(*π*(*t*)) or of *P*(*iω*) = *P*(*s*)|_*s* = *iω*_, with *τ* given by Eq (11). The second step is the time scale change *g*^−1^(*τ*) inverse to Eq (11). Since [*g*^−1^(*τ*)]^−1^ = *τ* = *g*(*t*), then the second step is

$$\begin{array}{c} \pi \left(t\right)=\frac{d}{dt}\left(g\left(t\right)\right)\pi^{C_{0}}\left(g\left(t\right)\right)=\frac{1}{N(t)}\pi^{C_{0}}\left(g\left(t\right)\right). \end{array}$$ (33)

It is obvious that in the first step we obtain probability distribution $\pi^{C_{0}}\left(\tau \right)$ which is the original of the Laplace transform *P*(*s*) or Fourier transform *P*(*iω*) and corresponds to the constant population size scenario with *N*_0_ = 1, while in the second step, by the time scale change *t* = *g*^−1^(*τ*) we obtain probability distribution *π*(*t*) under the scenario of the population size change given by *N*(*t*).

Using Eq (30) we can write expression for probability distribution of time *T*_*k*_ in the following integral form

πTk(t)=1N(t)12π∫-∞∞∏j=kn(j2)iω+(j2)exp(iω∫0tdσN(σ))dω, (34)

valid for the general case of the population size history *N*(*t*).

For the case of the exponential scenario of time change of the population size, the two steps mentioned above would assume the following forms. In the first step we compute probability distribution $\pi_{Tk}^{C_{0}}\left(\tau \right)$, of time to coalescence *T*_*k*_ under constant population size scenario with *N*_0_ = 1

πTkC0(τ)=12π∫-∞∞∏j=kn(j2)iω+(j2)exp(iωτ)dω. (35)

In the second step we transform $\pi_{Tk}^{C_{0}}\left(\tau \right)$ using Eq (11), which leads to

$$\begin{array}{c} t=g^{-1}\left(\tau \right)=\frac{1}{r}\ln\left(1+N_{0}r\tau \right), \end{array}$$ (36)

and

$$\begin{array}{c} \pi_{Tk}^{E}\left(t\right)=\frac{\exp(rt)}{N_{0}}\pi_{Tk}^{C_{0}}\left(\tau \left(t\right)\right)=\frac{1+N_{0}r\tau \left(t\right)}{N_{0}}\pi_{Tk}^{C_{0}}\left(\tau \left(t\right)\right). \end{array}$$ (37)

Distributions $\pi_{Tk}^{C_{0}}\left(\tau \right)$, Eq (35) and $\pi_{Tk}^{E}\left(t\right)$, Eq (37) are computed numerically. Numerical computations can be done in two ways, by numerical integration procedures, according to Eq (30), separately for each time point, or by using the inverse fast Fourier transform algorithm. In our computational examples, presented further in this paper, we have used both these approaches. For numerical integration we have used adaptive Gauss-Kronrod quadrature procedure [40] implemented as the Matlab function “quadgk”. Advantage of using numerical integration is that the time points can be freely located according to needs, which leads to lower errors. The disadvantage of the method of numerical integration is that it is slower compared to the fast Fourier transform algorithm.

The advantage of the fast Fourier transform algorithm is that it its much faster. However, estimating and controlling accuracy is more difficult. Despite problems with controlling accuracy, in the majority of computational examples we have computed Fourier integrals in Eq (35) by using Matlab inverse fast Fourier transform function “ifft”, taking advantage of its speed. In more detail, at first we have defined the time axis range and grid for $\pi_{Tk}^{C_{0}}\left(\tau \right)$ by using information on the first and second moments of $T_{k}^{C_{0}}$ [16] and assuming some additional margin related to skewness of the distributions. Time axis range and grid allows for defining the corresponding frequency axis ranges and grid and for computing $\pi_{Tk}^{C_{0}}\left(\tau \right)$ by the inverse fast Fourier transform procedure. We have estimated accuracy of computations by comparing known values of moments of times to values computed on the basis of numerically obtained distributions. In this way we have estimated that a grid with 500 equidistant time points was sufficient for obtaining relative error ≤ 10^−4^ for the case of computations for constant population size for *n* ≤ 10^4^. Nonlinear transformations of the time scale, necessary for computations for population exponential growth scenarios, result in nonuniformity of the time axis grid resolution, which leads to increase of the error. For the case of exponential scenarios of population growth we have experimentally verified that a grid with 1000 equidistant time points for *τ* was sufficient for obtaining relative error ≤ 10^−3^ for moments of distribution with the transformed time, with *n* ≤ 10^4^ and *ρ* ≤ 10^4^.

As supporting files (S1 File) to this paper we provide Matlab functions and scripts for computing probability distributions of times in the coalescence tree for exponential scenario of population growth, based on the direct method of numerical integration. We have tested these functions for the range of values of the product parameter 0 ≤ *ρ* ≤ 10^6^ and genealogy sizes *n* < = 10^4^. In the provided programs all parameters are set automatically and the relative errors are 10^−5^ or better.

### Limit distributions

According to our best knowledge no results concerning limit distributions of times close to the root of the coalescence tree, in particular *TMRCA*, were published in the literature. In this subsection we compute limit distributions for *TMRCA* for both constant and time varying population size scenarios. Denote the limit distribution of *TMRCA* under the population size scenario *N*(*t*) by *π*_*TMRCA*,∞_(*t*). On the basis of results from the previous subsection we have

πTMRCA,∞(t)=1N(t)12π∫-∞∞∏j=2∞(j2)iω+(j2)exp(iω∫0tdσN(σ))dω. (38)

Infinite product, which appears on the right hand side of the above formula can be analytically computed by using the following well known identity involving quotients of gamma functions (e.g., [21])

$$\begin{array}{c} \prod_{k=0}^{\infty }\frac{\left(k+a_{1}\right)\left(k+a_{2}\right)}{\left(k+b_{1}\right)\left(k+b_{2}\right)}=\frac{\Gamma \left(b_{1}\right)\Gamma \left(b_{2}\right)}{\Gamma \left(a_{1}\right)\Gamma \left(a_{2}\right)}, \end{array}$$ (39)

where Γ(.) denotes Euler’s gamma function and *a*_1_, *a*_2_, *b*_1_, *b*_2_ are any complex numbers satisfying *a*_1_ + *a*_2_ = *b*_1_ + *b*_2_. Using Eq (39) with *a*_1_ = 1, *a*_2_ = 2, $b_{1,2}=1.5\pm \sqrt{\frac{1}{4}-2i\omega }$, $i=\sqrt{-1}$, allows for deriving the following expression for the infinite product in Eq (38)

$$\begin{array}{c} \prod_{j=2}^{\infty }\;\frac{j(j-1)}{2i\omega +j(j-1)}=\frac{2\pi i\omega }{\cos[\pi \sqrt{\frac{1}{4}-2i\omega }]}. \end{array}$$ (40)

The above identity is listed in A. Dieckmann’s internet collection of infinite products [41]. Subsituting the above identity in Eq (38) one can compute the limit distribution of *TMRCA*, *π*_*TMRCA*,∞_(*t*) as follows

$$\begin{array}{c} \pi_{TMRCA,\infty }\left(t\right)=\frac{1}{N(t)}\frac{1}{2\pi }\int_{-\infty }^{\infty }\frac{2\pi i\omega }{\cos[\pi \sqrt{\frac{1}{4}-2i\omega }]}\exp\left(i\omega \int_{0}^{t}\frac{d\sigma }{N(\sigma )}\right)d\omega . \end{array}$$ (41)

We have numerically computed limit distribution *π*_*TMRCA*,∞_(*t*) given by the above formula Eq (41) using the direct method of numerical integration. When trying to apply fast Fourier transform algorithm we have encountered problems with the proper control of the accuracy of computations.

### Round-off errors in computing allelic frequencies

We have conducted a computational study on effects of round-off errors on accuracy of computation of expected allele frequencies by using expression Eq (24). Results in this subsection can be useful for such researches as those reported in [37], [38] and [16].

We denote the computed and the true expected allele frequencies by $f_{nb}^{comp}$ and $f_{nb}^{true}$ respectively, and we define the maximum relative error commited when computing allele frequencies, *MxRelErr*, as follows

$$\begin{array}{c} MxRelErr=\max_{{}_{{}_{1\leq b\leq n-1}}}\left|\frac{f_{nb}^{comp}-f_{nb}^{true}}{f_{nb}^{true}}\right|. \end{array}$$ (42)

By “computed allele frequencies” we mean values obtained by using expressions Eqs (24) and (25). One can estimate upper bound for *MxRelErr* by using error analysis technique. We define $f_{nb}^{comp}\left(\sigma \right)$ as representing values computed by using Eqs (24) and (25) in the case where expected times *e*_*j*_ in Eq (24) are additionally corrupted by Gaussian, relative error with standard deviation *σ*. By assuming the value of *σ* of one or two orders of magnitude higher than true relative round-off errors in computing *e*_*j*_ we can obtain the following, conservative, upper bound on *MxRelErr*

$$\begin{array}{c} MxRelErr<\max_{{}_{{}_{1\leq b\leq n-1}}}\left|\frac{f_{nb}^{comp}\left(\sigma \right)-f_{nb}^{comp}}{f_{nb}^{comp}}\right|. \end{array}$$ (43)

**Fig 7:**
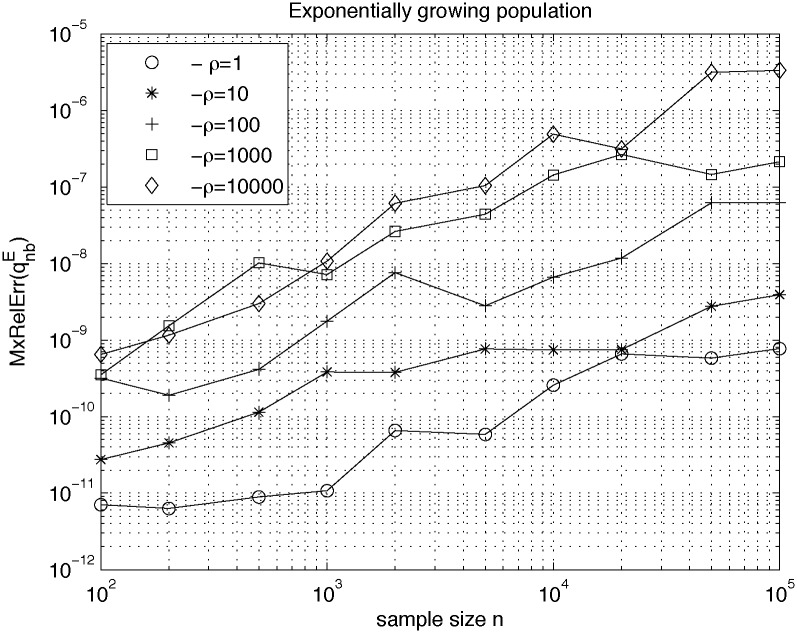
Influence of round-off errors on accuracy of computation of expected allele frequencies by using expressions Eqs (22)–(25). The plot shows upper bounds of maximum relative error for the scenario of exponential growth of population with different values of product parameter *ρ*, obtained by corrupting values of expected times *e*_*j*_ by Gaussian, relative error with standard deviation *σ* = 10^−13^.

In Fig 7 we show upper bounds of *MxRelErr* for the scenario of exponential growth of population with different values of product parameter *ρ*, obtained by assuming *σ* = 10^−13^. The assumed value of *σ* is approximately of one-two orders of magnitude higher than accuracy of computing $e_{j}^{E}$ in Eq (21), which we estimate to be in the range 10^−14^ − 10^−15^. Values of $e_{j}^{E}$ were computed by using Matlab function “expint” with the modification described in [8], which allows for obtaining exact function values for wide ranges of argument values.

Contemplating values of upper bounds for *MxRelErr* computed by using Eq (43), shown in Fig 7, we come to the conclusion that formulae Eqs (24) and (25) for computing allele frequencies *f*_*nb*_ can be safely used for sample sizes *n* up to the range of hundreds of thousands and expect relative errors not higher than 10^−6^.

Formulas for distributions of times in the coalescence tree, Eqs (31) and (32) can be used for computing expectations of coalescence times by numerical integration, which can be then substituted in Eq (3). This provides alternative method for computing allelic frequencies. Due to errors in numerical integration, higher by approximately two orders of magnitude than errors in computing values of special functions *Ei*(*x*), maximal relative round-off errors of allelic frequencies obtained by using Eq (3) and numerically computed expectations of coalescence times are in the range 10^−4^ − 10^−3^. They are significantly higher than those in Fig 7 but still acceptable in many applications.

## Supporting information

S1 File Software for computing probability distributions of times in the coalescence process. Archive with Matlab functions and scripts for computing probability distributions of times in the coalescence tree for exponential scenario of population growth, based on the direct method of numerical integration. Our Matlab functions and scripts are also available as a GitHub repository (https://github.com/agnieszkaszczesna/Coalescence-Computations-for-Large-Samples).
(ZIP)
